# Interplay among sex, environment, and heart-brain function in the onset of psychiatric disorders

**DOI:** 10.1192/j.eurpsy.2025.136

**Published:** 2025-08-26

**Authors:** E. Maggioni, P. Brambilla

**Affiliations:** 1 Dept. of Neurosciences and Mental Health, Fondazione IRCCS Ca’ Granda Ospedale Maggiore Policlinico; 2 Dept. of Electronics Information and bioengineering, Politecnico di Milano; 3 Dept. of Pathophysiology and Transplantation, University of Milan, Milan, Italy

## Abstract

**Abstract:**

Despite the crucial need for objective diagnostic procedures in psychiatry, the research of the quantitative neural bases of mental disorders is in its infancy, and currently there are no well-validated markers that could be translated into clinical practice. Candidate brain markers for neurodevelopmental, psychotic, and mood disorders have been proposed, but confounding factors and low statistical power leave most of these markers at the very early stage of development. Increasing evidence suggests a crucial role of biological, especially including sex, and environmental factors in shaping behavioral development and psychopathological risk. Moreover, such risk seems associated with greater susceptibility to cardiovascular problems, possibly mediated by alterations in the brain-heart axis. In this context, the study of brain maturation trajectories and brain-heart interactions in relation to sex and environment can provide key insights on the etiology of complex mental illnesses.

This lecture will provide an overview of our recent research on the interactions among biological factors, in particular sex, environmental risk, brain morphology and function, and cardiac autonomic regulation in affective and psychotic disorders using multivariate analysis approaches. Evidence obtained from juvenile cohorts, including samples of twins, will be presented to provide useful information on the genetic and environmental determinants of behavioral developmental trajectories, and on sex differences in these trajectories. The complex relationships among sex, environmental risk, autonomic regulation, brain morphology and connectivity, and mental and physical health will be explored in transdiagnostic samples of young adults and elders. Special focus will be given to sex-shared and sex-specific brain and autonomic mechanisms affected by social stressors, including discrimination, bullyism, and chronic stress, and their possible role in determining the heterogeneous clinical dimensions of psychotic and affective disorders.

**Disclosure of Interest:**

None Declared

